# TRIP: a method for novel transcript reconstruction from paired-end RNA-seq reads

**DOI:** 10.1186/1471-2105-13-S18-A11

**Published:** 2012-12-14

**Authors:** Serghei Mangul, Adrian Caciula, Dumitru Brinza, Ion I Mandoiu, Alex Zelikovsky

**Affiliations:** 1 Computer Science Department, Georgia State University, University Plaza, Atlanta, Georgia 30303, USA; 2 Ion Bioinformatics, Life Technologies Corporation, Foster City, CA, USA; 3 Department of Computer Science & Engineering, University of Connecticut, 371 Faireld Rd., Unit 2155, Storrs, CT 06269-2155, USA

## Background

Recent advances in DNA sequencing have made it possible to sequence the whole transcriptome by massively parallel sequencing, commonly referred as RNA-Seq. RNA-Seq is quickly becoming the technology of choice for transcriptome research and analyses. RNA-Seq allows to reduce the sequencing cost and significantly increase data throughput, but it is computationally challenging to use such RNA-Seq data for reconstructing of full length transcripts and accurately estimate their abundances across all cell types. A number of recent works have addressed the problem of transcriptome reconstruction from RNA-Seq reads. These methods fall into three categories: genome-guided, genome-independent and annotation-guided.

## Methods

In this work, we propose a novel statistical genome-guided method called “**T**ranscriptome **R**econstruction using **I**nteger **P**rograming” (TRIP) that incorporates fragment length distribution into novel transcript reconstruction from paired-end RNA-Seq reads. To reconstruct novel transcripts, we create a splice graph based on inferred exon boundaries and RNA-Seq reads. A splice graph is a directed acyclic graph (DAG), whose vertices represent exons and edges represent splicing events. We enumerate all maximal paths in the splice graph using a depth-first-search (DFS) algorithm. These paths correspond to putative transcripts and are the input for the TRIP algorithm.

To solve the transcriptome reconstruction problem we must select a set of putative transcripts with the highest support from the RNA-Seq reads. We formulate this problem as an integer program. The objective to select the smallest set of putative transcripts that yields a good statistical fit between the fragment length distribution empirically determined during library preparation and fragment lengths implied by mapping read pairs to selected transcripts.

## Conclusions

Preliminary experimental results on synthetic datasets generated with various sequencing parameters and distribution assumptions show that TRIP has increased transcriptome reconstruction accuracy compared to previous methods that ignore fragment length distribution information.

